# A Synbiotic of Lacto-*N*-tetraose and *Bifidobacterium animalis* subsp. *lactis* MN-Gup Attenuates High-Fat Diet-Induced Obesity by Modulating Metabolism and Gut Microbiota in Mice

**DOI:** 10.3390/nu18111681

**Published:** 2026-05-24

**Authors:** Ying Luo, Yang Li, Linjun Wu, Xiaoqiong Li, Xiangyu Bian, Jian Kuang, Jianqiang Li, Fangshu Shi, Xuguang Zhang, Xiaoqiang Han, Jinzhu Pang, Jinjun Li, Haibiao Sun

**Affiliations:** 1 First Clinical Medical College, Shanxi Medical University, Taiyuan 030000, China; 18617036291@163.com (Y.L.); jack98193@163.com (X.H.); 2 State Key Laboratory for Quality and Safety of Agro-Products, Institute of Food Sciences, Zhejiang Academy of Agricultural Sciences, Hangzhou 310021, China; 2112005190@zjut.edu.cn (L.W.); lixiaoqiong@zaas.ac.cn (X.L.); bianjinfeng1993@163.com (X.B.); jian_kuang95@yeah.net (J.K.); jianqiang.li910@outlook.com (J.L.); shifangshu1992@163.com (F.S.); 3 Academy of Medical Sciences, Shanxi Medical University, Taiyuan 030000, China; 4 Mengniu Institute of Nutrition Science, Global R&D Innovation Center, Inner Mongolia Mengniu Dairy (Group) Co., Ltd., Beijing 101107, China; liyang117112@mengniu.cn (Y.L.); fxgzhang@mengniu.cn (X.Z.)

**Keywords:** Lacto-*N*-tetraose, *Bifidobacterium animalis* subsp. *lactis* MN-Gup, synbiotics, gut microbiota, obesity

## Abstract

**Background/Objectives:** Obesity is closely associated with gut microbiota dysbiosis, intestinal barrier dysfunction, and impaired glucose and lipid metabolism. However, single probiotic or prebiotic interventions often yield only limited metabolic improvements. This study aimed to evaluate the effects of a synbiotic formulation comprising Lacto-*N*-tetraose (LNT) and *Bifidobacterium animalis* subsp. *lactis* MN-Gup (MN-Gup) in a high-fat diet (HFD)-induced obese mouse model. **Methods:** In this study, an HFD-induced obese mouse model was used to investigate whether the synbiotic formulation of LNT and MN-Gup could ameliorate obesity-related metabolic dysregulation, intestinal barrier dysfunction, and gut microbiota imbalance. Mice were treated with LNT alone, MN-Gup alone, or the synbiotic at different doses. Serum biochemical parameters, glucose tolerance, lipid profiles, liver histopathology, intestinal barrier markers, gut microbiota composition, short-chain fatty acid (SCFA) levels were analyzed. **Results:** High-dose synbiotic intervention significantly outperformed single-component treatments in reducing weight gain, improving glucose tolerance and lipid profiles, and attenuating hepatic lipid accumulation and injury in mice. These metabolic changes were accompanied by improved markers of intestinal barrier integrity and modulation of gut microbiota composition, characterized by the enrichment of beneficial genera (e.g., *Akkermansia*, *Leuconostoc*, and *Alistipes*) alongside a reduction in obesity-associated taxa (including *Desulfovibrionaceae_unclassified*, *Colidextribacter*, *Helicobacter*, *Erysipelatoclostridium*, *Peptococcaceae_unclassified*, and *Firmicutes_unclassified*). Spearman correlation analysis revealed associative links between microbial alterations and host metabolic markers. **Conclusions:** Collectively, these findings suggest that the synbiotic formulation comprising high-dose LNT and MN-Gup offers potential benefits for managing high-fat diet-induced metabolic dysregulation in mice.

## 1. Introduction

Obesity has become an international public health crisis that goes far beyond personal health. According to the latest data, more than 1 billion people around the world are currently suffering from obesity, including about 880 million adults and 159 million children and adolescents [1]. It is projected that 3.8 billion people worldwide will be overweight or obese by 2050, making up over half of the adult population [2]. Particularly concerning is the rising trend in childhood and adolescent obesity since 1990, which signals the transfer of health risks to younger populations and foretells a long-term, heavy burden on global healthcare systems [3]. Excessive adipose tissue buildup is a hallmark of obesity, a chronic metabolic illness. In addition to directly causing problems in lipid metabolism and being closely associated with metabolic syndromes, including non-alcoholic fatty liver disease and type 2 diabetes mellitus, it also functions as an independent risk factor for additional ailments like sleep apnea syndrome and osteoarthritis [4,5]. As a result, obesity is increasingly becoming the most common form of nutritional disorder in many countries, with far-reaching and widespread implications.

The gut microbiota is implicated in the development of obesity and obesity-related diseases, according to an increasing amount of research [6,7]. Obesity is frequently associated with reduced gut microbiota diversity, increased intestinal permeability, enhanced lipopolysaccharide (LPS) translocation, and an exacerbated systemic inflammatory response, all of which impair immune system performance. Furthermore, obesity-induced dysbiosis alters the metabolic functions of gut microbes, disrupting host glucose and lipid metabolism and related signaling processes and promoting endotoxin accumulation [8,9,10]. This set of alterations indicates that gut microbiota is a key pathophysiologic factor in the emergence of obesity.

Recognizing this complex and close connection between gut microbiota and obesity and its complications, scientists are actively exploring new ways to intervene in obesity by regulating gut microecology [11,12,13]. Research has suggested that MN-Gup effectively counteracts high-fat diet (HFD)-induced obesity through multiple mechanisms, including regulating gut microbiota composition and short-chain fatty acid (SCFA) production, reducing inflammatory responses, and improving metabolic parameters such as blood glucose and blood lipids [14,15]. Moreover, MN-Gup has been shown to facilitate lipid catabolism and stimulate GLP-1 secretion, offering substantial benefits for metabolic management [16]. However, the in vivo efficacy of solitary probiotics is frequently constrained by the host’s indigenous intestinal microenvironment and limited colonization efficiency [17].

To overcome these limitations, it is crucial to strategically apply prebiotics, defined as specific substrates selectively utilized by host microorganisms to confer a health benefit. Human milk oligosaccharides (HMOs) are uniquely potent prebiotics known to drive the early colonization and proliferation of beneficial taxa, particularly *Bifidobacterium* [18]. Lacto-*N*-tetraose (LNT), one of the most abundant HMOs, not only specifically favors the robust growth of *Bifidobacterium* [19,20] but also exerts profound immunomodulatory and anti-adhesive effects against opportunistic pathogens [21]. In addition, LNT has a supportive effect on intestinal barrier integrity [22], which helps to enhance intestinal physical defenses and reduce the entry of harmful substances, such as endotoxin, into the blood circulation. With two or even more health benefits, these actions collectively represent several protective mechanisms of LNT in the prevention of obesity and related metabolic illnesses.

Despite the recognized health benefits of MN-Gup and LNT individually, it remains unknown whether their combined application as a synbiotic offers superior therapeutic effects against obesity compared to monotherapies. Building on the individual properties discussed above, the specific rationale for this pairing lies in their highly complementary mechanisms targeting the gut ecosystem and metabolic homeostasis. First, to overcome the poor in vivo persistence of solitary probiotics in a dysbiotic gut, LNT serves as a highly selective nutritional niche. This specific substrate is expected to support the gastrointestinal survival of the administered MN-Gup and robustly augment the targeted *Bifidobacterium* population [23]. Second, LNT was selected as the prebiotic component because HMOs, particularly LNT, are known to be highly selective carbon sources for *Bifidobacterium* species, promoting their growth and metabolic activity [24]. Third, the efficacy of individual probiotics such as MN-Gup is frequently limited by poor colonization and survival in a dysbiotic gut environment. Therefore, we hypothesized that combining LNT with MN-Gup would produce superior anti-obesity effects compared with either component alone, not by creating a formal synergistic interaction, but by addressing the limitations of each monotherapy through complementary mechanisms. Specifically, LNT is expected to provide an exclusive nutritional niche that enhances the gastrointestinal survival, proliferation, and colonization of MN-Gup. In turn, the augmented proliferation of MN-Gup is expected to accelerate the fermentation of LNT into SCFAs [25], which exert direct regulatory effects on hepatic lipid metabolism and trigger cross-feeding networks that enrich other beneficial taxa [26]. Furthermore, the combined intervention may fortify intestinal barrier integrity through complementary actions: LNT contributes through its structural properties [27], while MN-Gup contributes through its metabolic outputs (e.g., SCFAs) [28]. To test this hypothesis and address the current knowledge gap, the present study was designed to systematically evaluate the anti-obesity effects of the LNT+MN-Gup synbiotic formulation in an HFD-induced obese mouse model. A primary goal was to determine whether the combined intervention produces superior metabolic benefits compared to individual treatments. Specifically, we assessed its capacity to ameliorate macroscopic obesity phenotypes (e.g., body weight and adiposity), improve systemic glucolipid metabolism, and restore intestinal barrier integrity. Furthermore, we sought to characterize the underlying mechanisms driven by the remodeling of gut microbiota composition and its associated metabolic pathways. By integrating these physiological and microbiological findings, this study aims to provide a robust scientific foundation for utilizing this novel synbiotic combination as a precision nutritional strategy for obesity prevention and management.

## 2. Materials and Methods

### 2.1. Preparation and Administration of Bifidobacterium animalis subsp. lactis MN-Gup

The probiotic *Bifidobacterium animalis* subsp. *lactis* MN-Gup (CGMCC No. 15578), provided by China Mengniu Dairy Company Limited (Beijing, China), was cultivated in De Man, Rogosa, and Sharpe (MRS) culture medium for 24 h at 37 °C under anaerobic conditions using a large anaerobic workstation (ELECTROTEK, Aberdeen, UK). Following centrifugation (8000× *g*, 4 °C, 5 min) to extract the cultivated bacteria, they were resuspended in sterile phosphate-buffered saline (PBS) and then subjected to three rounds of washing. The suspension was diluted to a concentration of 10^9^ CFUs in 1 mL and then suspended in a sterile PBS solution for probiotic administration.

### 2.2. Animals, Diets, and Experimental Design

All experiments complied with the rules set forth by the Zhejiang Academy of Agricultural Sciences’ Ethics Committee on Animal Experiments (Source: 25ZALAS10). Male C57BL/6 mice weighing 22–23 g and 6–8 weeks of age were bought from GemPharmatech Co., Ltd. (Nanjing, China) and kept in the Animal Experimental Center of the Zhejiang Academy of Agricultural Sciences. After one week of acclimatization with free access to food and water, the mice were initially divided into two groups: one group (*n* = 7) was fed a standard chow diet as the normal control group (NC), and the remaining 35 mice were fed a 60% high-fat diet for eight weeks to induce obesity. After the 8-week feeding period, the HFD-fed mice were ranked by body weight and then assigned to five groups using a randomized block design to ensure baseline body weight consistency across groups. The five groups were the high-fat diet group (HFD), the probiotic group (MN-Gup), the prebiotic group (LNT), the low-dose synbiotic group (LNT+MN-Gup-L), and the high-dose synbiotic group (LNT+MN-Gup-H), with 7 mice in each group. Together with the NC group, a total of 42 mice were used in this study. Based on previous research [29], a sample size of *n* = 7 per group was chosen, which is adequate for identifying significant differences. To further confirm the adequacy, a post hoc power analysis was performed using G*Power software (version 3.1.9.7). Assuming a one-way ANOVA with six groups, a large effect size (f = 2.05, derived from pilot data), α = 0.05, and a desired power of 0.80, the required total sample size was calculated to be 12 mice. Using the actual sample size (*n* = 7 per group, total = 42), the achieved power exceeded 0.95. For eight weeks, mice in the NC group were fed a standard chow diet, while those in the remaining groups received a high-fat diet in which 60% of the total calories were derived from fat (Product XTHF60; Jiangsu Xietong Pharmaceutical Bio-engineering Co., Ltd., Jiangsu, China). Every day, mice in the NC and HFD groups were gavaged with 200 μL of regular saline. The mice in the probiotic group received MN-Gup (1 × 10^9^ CFU/day). This dosage was determined based on the effective dose of *Bifidobacterium animalis* subsp. *lactis* A6 for reducing obesity [30]. The prebiotic group received LNT at a dosage of 400 mg/kg/day. This dose was calculated by simulating the natural intake of human milk oligosaccharides: referencing the natural concentration of LNT in mature human milk (0.5 g/L) [31], the estimated intake for an infant (5 kg body weight consuming 800 mL daily) is 80 mg/kg/day, which converts to a mouse dose of 400 mg/kg/day using the body surface area (BSA) method [32]. The synbiotic group was divided into two subgroups: a low-concentration group (2.5 × 10^8^ CFU/day of MN-Gup + 100 mg/kg/day of LNT) and a high-concentration group (1 × 10^9^ CFU/day of MN-Gup + 400 mg/kg/day of LNT). The low LNT dose (100 mg/kg/day) was chosen as it is an established physiologically active dose in obesity models that effectively regulates gut microbiota and immune pathways [33,34]. The low probiotic dose (2.5 × 10^8^ CFU/day) was selected based on a systematic review by Álvarez-Arraño et al. [35], which reported that effective probiotic doses range from 10^6^ to 5 × 10^10^ CFU/day. All dosages were selected to align with effective ranges reported in previous studies for *B. lactis* and HMOs in murine models to evaluate a potential dose–response relationship. All treatments were given at a volume of 200 μL/mouse/day. For eight weeks, the medication was administered, with regular monitoring of the animal’s body weight, food, water, and fasting blood glucose (FBG). In this experiment, humane endpoints were defined as a weight loss of 15 percent or more relative to peak body weight. In order to collect tissue samples, the animals were then promptly put to death by overdosing on sodium pentobarbital (150 mg/kg, intraperitoneal injection). It is important to note that throughout the entire experimental duration, no pharmacological agents or additional glucose-regulating medications were administered to any of the experimental groups. All observed physiological and metabolic alterations were exclusively attributable to the defined dietary regimens and the specific prebiotic/probiotic interventions.

### 2.3. Intraperitoneal Glucose Tolerance Test (IPGTT)

The mice were fasted for 12 h before the glucose level was measured. Following an intraperitoneal injection of glucose (2 g/kg), blood was drawn from the tail vein. A blood glucose meter (Yuwell, Shanghai, China) was used to monitor the fasting blood glucose levels at 0, 15, 30, 60, and 120 min [36].

### 2.4. Serum Biochemical Analysis

The mice were kept in a fasting and water-free state for 12 h. Fresh orbital blood was then collected, centrifuged for 10 min at 4 °C at 4000 rpm, and stored long-term in a refrigerator at −80 °C. Following the manufacturer’s instructions, assay kits provided by Beijing Pulilai Gene Technology Co., Ltd. (Beijing, China) were used to measure the levels of total triglycerides (TG) and total cholesterol (TC) in mouse serum. Assay kits from Nanjing Jiancheng Bioengineering Institute (Nanjing, China) were used to assess the levels of alanine aminotransferase (ALT), aspartate aminotransferase (AST), high-density lipoprotein cholesterol (HDL-C), and low-density lipoprotein cholesterol (LDL-C) in mouse serum. Jiangsu Jingmei Biological Technology Co., Ltd. (Yancheng, China) supplied the mouse leptin (LEP) and insulin enzyme-linked immunosorbent assay (ELISA) kits, which were used in accordance with the guidelines. All operations were performed according to the instructions. Homeostatic model assessment of insulin resistance (HOMA-IR) was calculated as fasting glucose × fasting insulin/22.5.

### 2.5. Hepatic Function Tests

A SCIENTZ-48 tissue grinder (Ningbo Scientz Biotechnology Co., Ltd., Ningbo, China) was used to mince, homogenize, and centrifuge the liver tissue at 2000× *g* for 10 min. Subsequently, the cleared supernatant was collected. The levels of TC, TG, total superoxide dismutase (T-SOD), and glutathione peroxidase (GSH-PX) in the liver tissue were measured using kits (Nanjing Jiancheng Bioengineering—Nanjing, China, and Beijing Pulilai Gene Technology Co., Ltd.). The homogenized supernatant was used to assess liver function and lipid indices. Each step was performed according to the kit’s instructions.

### 2.6. Histological Analysis

Although the murine cecum represents a major site of microbial fermentation, colonic tissue was specifically selected for subsequent histological and molecular analyses. This strategic selection was based on the colon’s superior translational relevance to human gastrointestinal physiology. For histological evaluation, the collected colon, liver, and epididymal adipose tissues were fixed in 4% paraformaldehyde (PFA). The fixed tissues were sent to Wuhan Servicebio Biotechnology Co., Ltd. (Wuhan, China) on dry ice, where they were fixed for 24 h, embedded in paraffin, sliced into 3 µm sections, and stained with hematoxylin and eosin (H&E) (Sigma-Aldrich, St. Louis, MO, USA). Additionally, Oil Red O staining was performed to analyze changes in liver fat deposition. The area that was stained red by Oil Red O was then calculated and divided by the section’s overall area. Ultimately, the result was given as a percentage of the area of the section stained positively for Oil Red O. To avoid observer bias, tissue sections were randomized and given numerical codes before microscopic evaluation. The investigator performing the histological scoring was blinded to the treatment groups until all analyses were completed.

### 2.7. 16S rRNA Sequencing

The 16S rRNA amplicon sequencing was performed and analyzed by LC-Bio Technologies Co., Ltd. (Hangzhou, China). Using region-specific primers, the bacterial 16S rRNA gene’s V3–V4 hypervariable regions were amplified. The raw sequencing data were initially processed by merging paired-end reads based on their overlapping regions, followed by filtering low-quality sequences and chimeras to obtain high-quality clean data. Amplicon Sequence Variants (ASVs) and their associated abundance table were then produced after the clean data had been processed through the QIIME2 workflow and sequence denoising had been carried out using the DADA2 plugin to eliminate any possible PCR and sequencing errors. The sequencing depth was approximately 85,000 reads per sample. The rarefaction curves for all samples are provided in the Supplementary Materials. These data were then used for downstream analyses, including the assessment of alpha diversity (using Chao1, observed species, Shannon, and Simpson indices) and beta diversity (visualized via non-metric multidimensional scaling based on Bray–Curtis dissimilarity). The NCBI and SILVA reference databases were used for taxonomic classification in order to examine the phylum- and genus-level microbial composition. Differentially abundant taxa between groups were identified using linear discriminant analysis effect size (LEfSe), and PICRUSt2 was used to infer functional pathways based on the 16S rRNA profiles. The 16S rRNA sequencing data were deposited in the NCBI Sequence Read Archive (SRA) under the BioProject accession number PRJNA1394527. The 16S rRNA sequencing data will be made publicly available upon publication of this article.

### 2.8. Short-Chain Fatty Acids (SCFAs) Analysis

SCFAs were determined by GC-FID with reference to the method reported by Liao et al. [37], with modifications for fecal samples. Fecal samples were collected from mice and immediately stored at −80 °C until analysis. For SCFA determination, approximately 100 mg of each fecal sample was suspended in 1.0 mL of ultrapure water, vortexed thoroughly, and homogenized on ice. The suspension was acidified with 50 μL of 50% sulfuric acid to ensure that the pH was below 3.0, followed by centrifugation at 12,000× *g* for 10 min at 4 °C. The supernatant was collected and filtered through a 0.22 μm membrane prior to chromatographic analysis. SCFAs were quantified by gas chromatography using a GC9720II gas chromatograph (Fuli Analytical Instrument Co., Ltd., Taizhou, China) equipped with a flame ionization detector (FID) and an Agilent-FFAP capillary column (30 m × 0.25 mm × 0.25 μm). The injection volume was 1.0 μL, and each sample was analyzed in triplicate. The injector temperature was set at 220 °C. The oven temperature program was as follows: initial temperature 75 °C; increased to 180 °C at 20 °C/min and held for 1 min; then increased to 220 °C at 50 °C/min and held for 1 min. High-purity nitrogen was used as the carrier gas at a flow rate of 2.5 mL/min, and the split ratio was 5:1. The detector temperature was maintained at 250 °C. Acetate, propionate, isobutyrate, butyrate, isovalerate, and valerate were identified and quantified using authentic external standards. SCFA concentrations were calculated from standard calibration curves and expressed as μmol/g wet feces.

### 2.9. RNA Preparation and Gene Expression Analysis by Real-Time qPCR

Total RNA was isolated from colon, liver, and epididymal adipose tissues using Trizol reagent. First-strand cDNA was synthesized from 1 μg of total RNA using the TransScript All-in-One First-Strand cDNA Synthesis Kit (TransGen Biotech, Beijing, China) under the following conditions: 42 °C for 15 min, followed by 85 °C for 5 s. Quantitative real-time PCR (qPCR) was performed on a Bio-Rad CFX96 system (Bio-Rad Laboratories, Hercules, CA, USA) using SYBR Green Master Mix. The reactions were carried out in a 20 μL volume containing 10 μL of 2× SYBR Green Mix, 0.4 μL of each primer (10 μM), 2 μL of cDNA, and 7.2 μL of nuclease-free water. The thermal cycling conditions were 94 °C for 30 s, followed by 40 cycles of 94 °C for 5 s and 60 °C for 30 s. *Gapdh* was used as the internal control to normalize gene expression levels. Relative quantification was calculated using the 2^−ΔΔCt^ method. All primer pairs were synthesized by Tsingke Biotechnology (Beijing, China), and the sequences are shown in Table 1.

### 2.10. Statistical Analysis

The mean ± standard error (SEM) was used to express the data from at least three independent experiments. GraphPad Prism was used to analyze the data (GraphPad Software 10.0). The Shapiro–Wilk test was used to determine normality for comparisons involving more than two groups, and the Brown–Forsythe test was used to validate homogeneity of variances. Tukey’s post hoc test for multiple comparisons was then performed after one-way analysis of variance (ANOVA). The non-parametric Kruskal–Wallis test and Dunn’s post hoc test were used if these assumptions were violated. An unpaired Student’s *t*-test (two-tailed) was employed for comparisons between two groups if the data were normally distributed; otherwise, the non-parametric Mann–Whitney U test was utilized. Spearman correlation analysis was used to evaluate the relationship between the relative abundance of bacterial genera and basic parameters. For Spearman correlation analysis, *p* values were adjusted for multiple comparisons using the Benjamini–Hochberg method to control the false discovery rate (FDR). Differences and correlations were considered statistically significant when *p* < 0.05.

## 3. Results

### 3.1. LNT+MN-Gup-H Significantly Reduces Weight Gain and Improves Glucose Tolerance and Plasma Biochemical Profile

**Figure 1:**
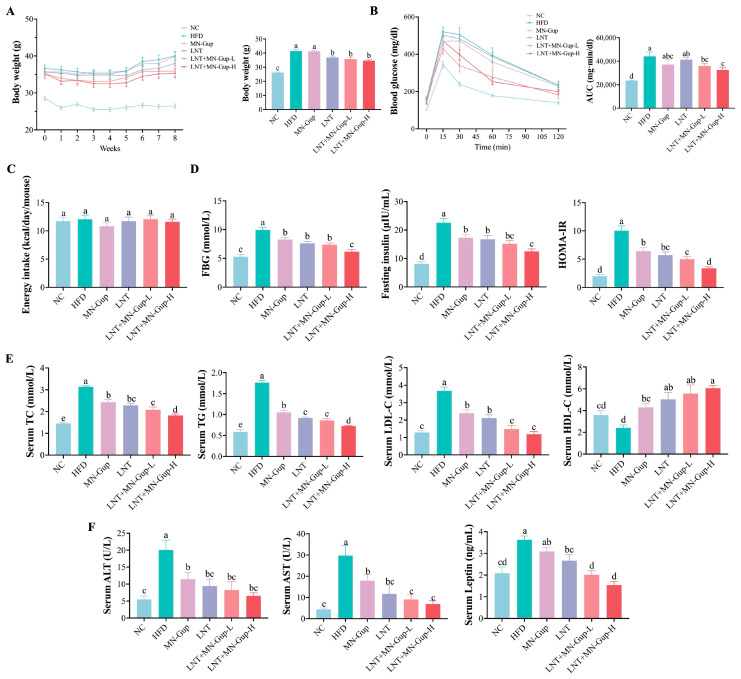
Effects of synbiotic administration on (**A**) body weight; (**B**) glucose tolerance test and area under the curve (AUC); (**C**) Energy intake; (**D**) FBG, fasting insulin levels, and HOMA-IR; (**E**) Serum TC, TG, HDL-C and LDL-C levels; (**F**) Serum ALT, AST and Leptin levels. Data are expressed as mean ± SEM (*n* = 7). Different lowercase letters indicate significant differences among groups at *p* < 0.05.

During the 8-week treatment period, the body weights of all experimental group mice were recorded weekly at fixed intervals. The results showed that compared to the NC group, the HFD group exhibited a significant increase in body weight, whereas synbiotic intervention significantly attenuated this trend of HFD-induced weight gain. At the experimental endpoint, intervention with LNT+MN-Gup was associated with a reduction in body weight relative to the HFD group (Figure 1A). During the IPGTT, the NC group exhibited a normal physiological glucose clearance trajectory, characterized by a rapid return to baseline following a transient initial peak. In stark contrast, the HFD group exhibited severe glucose intolerance with sustained hyperglycemia. Against this pathological background, the IPGTT data suggested that LNT+MN-Gup was associated with improved glucose homeostasis in HFD mice. Specifically, LNT+MN-Gup treatment markedly alleviated blood glucose fluctuations, as evidenced by the significant decrease in AUC values in both LNT+MN-Gup-treated groups relative to the HFD group (Figure 1B). This indicates that the synbiotic can improve glucose metabolic imbalance in HFD mice. Consistent with the IPGTT results, measurements of fasting metabolic parameters further supported the beneficial effects of LNT+MN-Gup on glucose homeostasis. First, energy intake analysis revealed no significant differences among the HFD-fed groups (*p* > 0.05), suggesting that the improvements in body weight and metabolic parameters were attributable to metabolic modulation induced by the synbiotic intervention rather than reduced caloric intake (Figure 1C). Furthermore, compared with the HFD group, LNT+MN-Gup intervention significantly reduced fasting blood glucose (FBG) and fasting insulin levels (*p* < 0.05), accompanied by a marked decrease in the HOMA-IR index (*p* < 0.05), indicating a potential improvement in insulin sensitivity (Figure 1D). Figure 1E,F illustrate the effects of LNT+MN-Gup administration on serum lipids and liver function. Analysis revealed that LNT+MN-Gup intervention significantly reduced the serum levels of AST, ALT, TC, TG, LDL-C, and LEP, while markedly elevating HDL-C levels, with the synbiotic treatment demonstrating a distinct advantage over single-component groups. Notably, in terms of regulating serum LDL-C, TC, TG, and leptin levels, the synbiotic treatment showed trends toward greater improvement compared to single-component groups.

### 3.2. LNT+MN-Gup-H Significantly Alleviates Liver Injury and Lipid Accumulation

**Figure 2:**
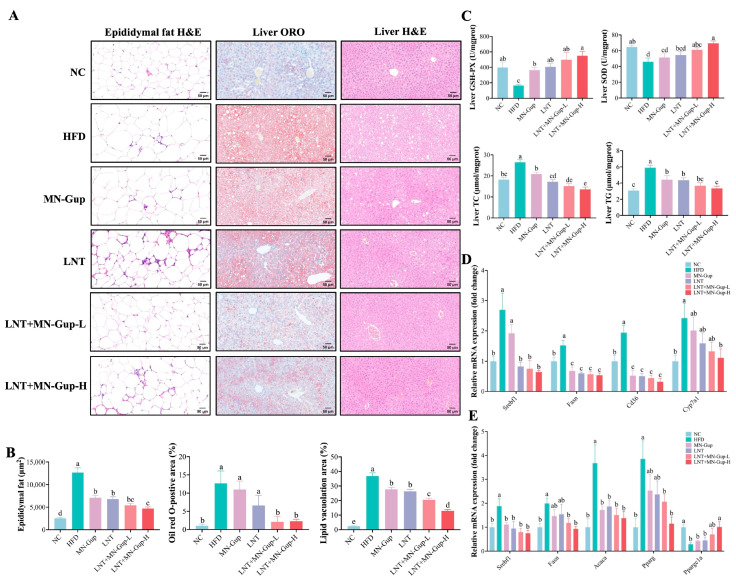
Synbiotics alleviate liver injury and lipid accumulation. (**A**) epididymal adipose tissue, stained with hematoxylin and eosin (scale bar = 50 µm); liver tissue, analyzed by Oil Red O staining (scale bar = 50 µm); liver tissue, analyzed by hematoxylin and eosin staining (scale bar = 50 µm); (**B**) Quantification of epididymal adipocyte size, hepatic Oil Red O-positive area, and hepatic lipid vacuolation area using ImageJ 1.54p software; (**C**) liver TC, liver TG, liver SOD, and liver GSH-PX; (**D**) gene expression of *Cyp7a1*, *Cd36*, *Srebf1*, *and Fasn* in the liver; (**E**) gene expression of *Srebf1*, *Acaca*, *Fasn*, *Pparg*, and *Ppargc1a* in epididymal fat. Data are expressed as means ± SEM (*n* = 7). Different lowercase letters indicate significant differences among groups at *p* < 0.05.

To assess hepatic lipid deposition, liver tissues from each group of mice underwent Oil Red O staining. Figure 2A presents the Oil Red O staining results illustrating lipid deposition in the liver tissues of each group. The figure demonstrates severe lipid deposition in the liver tissues of the HFD group, characterized by numerous large red lipid droplets exhibiting diffuse distribution. Following treatment with either the single component or the synbiotic, this phenotype showed improvement. The LNT+MN-Gup-H group exhibited the greatest observed reduction in both the number and size of red lipid droplets. Compared with the HFD group, the synbiotic-treated group showed significantly fewer and smaller red lipid droplets, along with a markedly lower histological score. To further evaluate hepatic histology, hematoxylin and eosin (H&E) staining was conducted on liver sections from all groups. H&E staining revealed marked lipid vacuole accumulation and pale cytoplasmic staining in hepatocytes of the HFD group. Both monotherapy and combined treatment significantly improved these pathological features. Following synbiotic therapy, hepatic lipid vacuoles were reduced and hepatocyte cytoplasmic staining improved, with the LNT+MN-Gup-H group showing the greatest effect among the tested groups. Quantitative analysis of liver H&E staining further confirmed these histopathological observations. Compared with the HFD group, both single-component and synbiotic interventions significantly reduced the corresponding histological score, with the synbiotic-treated groups showing a more marked improvement, particularly in the LNT+MN-Gup-H group (Figure 2B). Furthermore, to investigate the regulatory effects of synbiotics on hepatic metabolic function, biochemical parameter assessments were conducted. Figure 2C illustrates the effects of experimental interventions on hepatic lipid metabolism and oxidative stress status in HFD mice. Compared with the HFD group, single-component interventions significantly reduced TG levels and increased GSH-Px activity; these were the only significant changes observed. Synbiotic treatment was associated with reductions in hepatic TC and TG levels and concurrent increases in SOD and GSH-Px activity, with the LNT+MN-Gup-H group showing relatively greater improvements. To further investigate the impact on liver lipid metabolism-related gene expression, RT-PCR analysis was performed on liver tissue. Results revealed significantly elevated *Cyp7a1*, *Cd36*, *Srebf1*, and *Fasn* expression in the HFD group compared to the NC group. Single-component and synbiotic treatments were associated with improvements in these gene expression levels, with the LNT+MN-Gup-H group showing relatively greater changes. These findings suggest that synbiotics may modulate the expression of genes involved in fatty acid oxidation and lipid synthesis (Figure 2D). HE staining of epididymal fat revealed markedly enlarged adipocytes in the HFD group compared to the NC group, which showed noticeable improvement following synbiotic treatment. Quantitative analysis confirmed that synbiotic treatment significantly reduced adipocyte area, with the LNT+MN-Gup-H group exhibiting the optimal effect. To further investigate the intervention’s impact on lipid-regulating genes in epididymal adipose tissue, we examined relevant genes in this tissue. Results indicated that synbiotic treatment significantly restored *Srebf1*, *Acaca*, *Fasn*, and *Ppargc1a* gene expression in HFD mice, with relatively greater changes compared to single components. These observations may indicate that synbiotic treatment modulates the transcriptional profiles of genes involved in de novo lipogenesis and lipid oxidation, potentially contributing to the observed improvements in lipid metabolism (Figure 2E).

### 3.3. LNT+MN-Gup-H Significantly Ameliorates Intestinal Barrier Dysfunction

**Figure 3:**
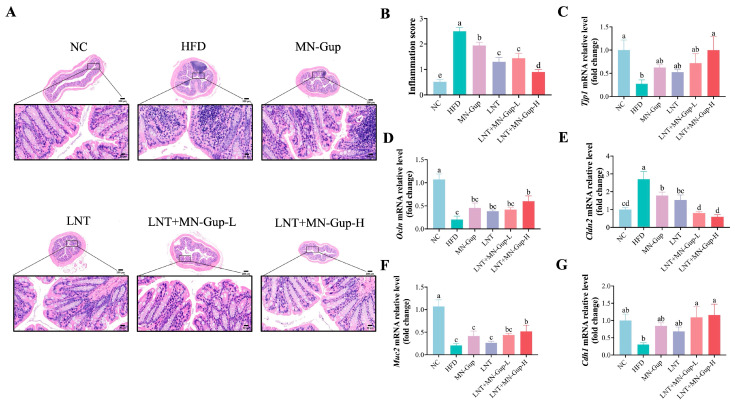
Effects of synbiotics administration on (**A**) colon tissue, analyzed by hematoxylin and eosin staining (scale bar = 20 µm); (**B**) Colonic inflammation score; (**C**–**G**) gene expression of intestinal barrier integrity markers *Tjp1*, *Ocln*, *Cdh1*, *Cldn2*, and *Muc2* in HFD-fed mice, analyzed by real-time qPCR. Data are expressed as means ± SEM (*n* = 7). Different lowercase letters indicate significant differences among groups at *p* < 0.05.

To assess the impact of the intervention on intestinal morphology and histological integrity, we analyzed intestinal tissue sections. Compared with the NC group, the HFD group exhibited extensive inflammatory cell infiltration and disruption of intestinal villus architecture, whereas the LNT+MN-Gup-H group was associated with improvement in HFD-induced intestinal damage, accompanied by reduced inflammatory cell infiltration (Figure 3A). The colonic inflammation scores were significantly higher in the HFD group compared with the NC group, whereas the scores in the MN-Gup, LNT, and LNT+MN-Gup-L groups were significantly lower than those in the HFD group. The LNT+MN-Gup-H group showed relatively lower scores compared to the MN-Gup, LNT, and LNT+MN-Gup-L groups, suggesting a trend toward improved colonic histology (Figure 3B). To further investigate the intervention’s impact on intestinal barrier integrity, we examined tight junction-related genes in mouse colonic tissue. The results suggested that the expression of genes vital for maintaining intestinal barrier architecture was significantly compromised by a high-fat diet. *Cldn2*, a gene associated with barrier permeability, was significantly up-regulated in the colonic tissues of the HFD group, while the expression of barrier integrity genes (e.g., *Tjp1*, *Ocln*, *Cdh1*, and *Muc2*) was significantly down-regulated compared to the NC group (Figure 3C–G). The expression of barrier integrity genes was restored, while *Cldn2* expression was decreased to varied degrees in the MN-Gup, LNT, and synbiotic groups compared to the HFD group. The LNT+MN-Gup-H group was associated with relatively greater changes in these genes compared to MN-Gup or LNT alone. These observations suggest that synbiotic therapy may contribute to improvements in intestinal histology and be associated with modulation of key tight junction-related genes, potentially supporting barrier function.

### 3.4. LNT+MN-Gup-H Modulates Gut Microbiota and SCFA Profiles

**Figure 4:**
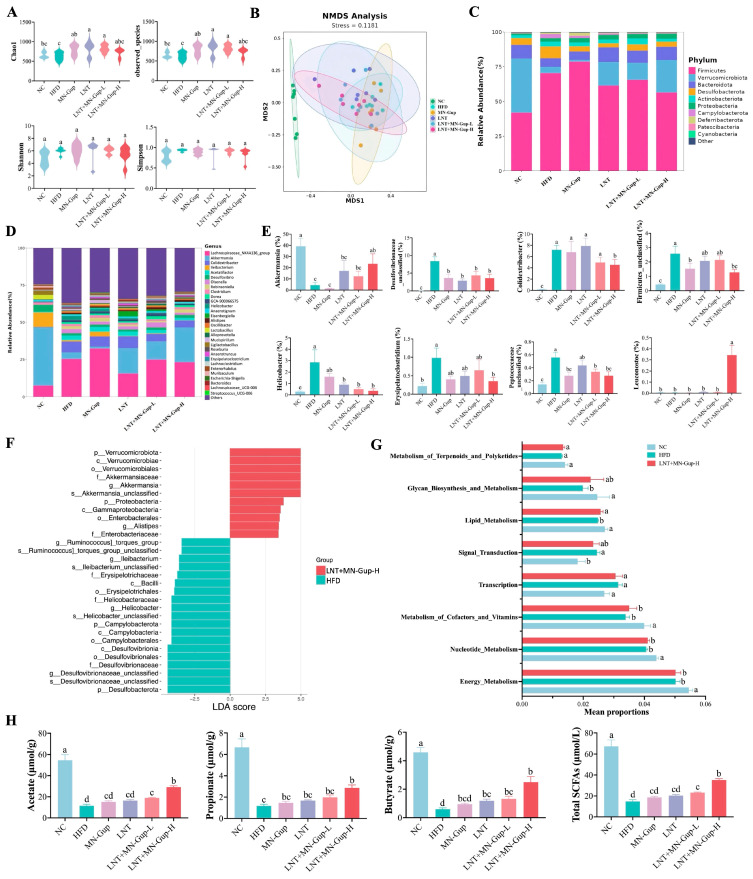
Impact of synbiotic administration on gut microbiota composition, functional prediction, and SCFA levels in HFD-induced obese mice. (**A**) Alpha diversity (Chao1, observed species, Shannon, and Simpson indices); (**B**) Beta diversity assessed by non-metric multidimensional scaling (NMDS); (**C**) Stacked bar plot of microbial community composition at the phylum level; (**D**) Stacked bar plot of microbial community composition at the genus level; (**E**) Differentially abundant bacterial genera among groups; (**F**) Cladogram of LDA values of biomarkers in the linear discriminant analysis effect size (LEfSe) analysis; (**G**) PICRUSt2 prediction of KEGG pathway categories at level 2; (**H**) Effect of synbiotic administration on SCFA levels in HFD-induced obesity, including acetate, propionate, butyrate, and total SCFAs. p, phylum; c, class; o, order; f, family; g, genus. Data are expressed as means ± SEM (*n* = 7). Different lowercase letters indicate significant differences among groups at *p* < 0.05.

Disruption of the gut microbiota is closely associated with obesity. Modulating the gut microbiota may be associated with improvements in metabolic homeostasis and energy balance in preclinical models, representing a potential avenue for obesity intervention. Analysis of α-diversity indices showed that the Chao1 and observed species indices tended to decrease in the HFD group compared with the NC group, although no significant difference was observed. MN-Gup, LNT, and synbiotic treatments increased richness relative to the HFD group, with some groups reaching statistical significance. In contrast, no significant differences were detected among groups in Shannon and Simpson indices (Figure 4A). Further non-metric multidimensional scaling (NMDS) based on Bray–Curtis distance matrices explored the impact of interventions on gut microbiota structure. Results indicated a marked divergence in microbiota structure between the HFD group and the NC group along the MDS1 dimension, reflecting significant compositional differences between these groups. The intervention groups exhibited only partial overlap with the HFD group and showed a tendency to shift toward the NC group, indicating partial rather than complete restoration of the gut microbiota structure. Notably, the LNT+MN-Gup-treated groups were positioned between the HFD and NC groups, consistent with partial remodeling (Figure 4B, stress = 0.1181). HFD feeding induced notable alterations in gut microbiota composition at both the phylum and genus levels. Intervention with MN-Gup, LNT, or their combination partially modulated these changes. The combined treatment groups exhibited a trend toward a microbiota composition closer to that of the NC group, although complete restoration was not observed. Relative abundance analysis at the phylum level showed that Firmicutes, Verrucomicrobiota, Bacteroidota, Desulfobacterota, Actinobacteriota, and Proteobacteria were the dominant phyla in each group (Figure 4C). Compared with the NC group, the HFD group exhibited a lower relative abundance of Verrucomicrobiota (4.54% vs. 39.12%) and a higher relative abundance of Firmicutes (70.49% vs. 42.00%), together with shifts in Bacteroidota (6.21% vs. 9.72%) and Desulfobacterota (8.48% vs. 4.82%). At the genus level, *Akkermansia* was significantly decreased in the HFD group (4.54% vs. 39.12%), whereas *Colidextribacter* (7.19% vs. <1.0%), *Desulfovibrionaceae_unclassified*, *Peptococcaceae_unclassified*, *Erysipelatoclostridium*, *Helicobacter*, and *Firmicutes_unclassified* were significantly increased (Figure 4D). These changes were partially reversed after intervention, with *Akkermansia* showing significant recovery and *Leuconostoc* showing a significant increase in the LNT+MN-Gup-H group (*p* < 0.05, Figure 4E). To further investigate the signature genera of the LNT+MN-Gup-H group, LEfSe analysis (LDA ≥ 3.0) was conducted. Results indicated that enriched biomarkers in this group included *Akkermansia* and *Alistipes* (Figure 4F). Based on 16S rRNA gene sequencing, PICRUSt2 was used to predict the functional potential of the gut microbiota according to the KEGG database. The results showed that the predicted functions were mainly enriched in metabolism-related pathways at KEGG level 2. Among these, lipid metabolism exhibited significant differences among groups, with the HFD group showing a lower relative abundance compared with the NC group, while the LNT+MN-Gup-H group was associated with an increased relative abundance compared with the HFD group. In addition, energy metabolism, nucleotide metabolism, and metabolism of cofactors and vitamins also showed differences among groups, generally displaying lower relative abundance in the HFD group and higher levels in the intervention group. Glycan biosynthesis and metabolism showed a similar pattern, while signal transduction varied among groups without a consistent trend. In contrast, transcription and metabolism of terpenoids and polyketides showed relatively minor differences across groups (Figure 4G). HFD feeding significantly decreased the levels of acetate, propionate, butyrate, and total SCFAs compared with the NC group. Both MN-Gup and LNT treatments increased SCFA levels to varying extents, while the combined intervention further elevated these levels. Among the intervention groups, LNT+MN-Gup-H generally exhibited higher SCFA levels than the other treatment groups. However, SCFA levels in all intervention groups remained lower than those in the NC group (Figure 4H). Importantly, these microbiome and SCFA findings should be interpreted as associative and exploratory. Because PICRUSt2 relies on 16S rRNA gene profiles, the identified metabolic pathway shifts are strictly predictive. Furthermore, while the restoration of SCFA profiles aligns with the observed phenotypic improvements, these data do not establish a direct causal relationship.

### 3.5. Spearman Correlation Analysis Between Bacterial Genus and Basic Indicators

**Figure 5:**
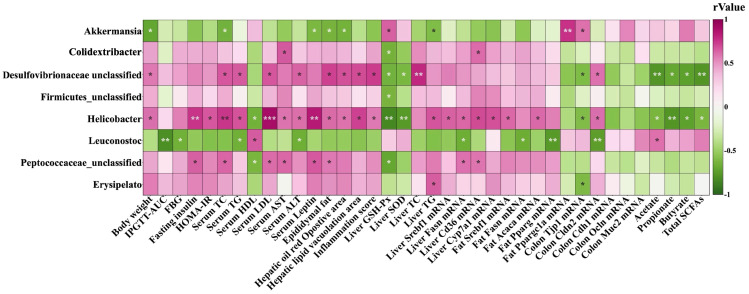
Spearman correlation analysis between bacterial genus and basic indicators. Correlations were considered statistically significant when * *p* < 0.05, ** *p* < 0.01, and *** *p* < 0.001.

Spearman correlation analysis showed that obesity-related indices, including body weight, serum lipids, liver function parameters, and lipid metabolism-related gene expression, were generally positively correlated with each other, whereas SCFA-related indices showed opposite correlation patterns with most metabolic disorder indicators (Figure S3). At the microbial level, *Akkermansia* was generally negatively correlated with obesity-related parameters, while *Colidextribacter*, *Desulfovibrionaceae_unclassified*, and *Erysipelatoclostridium* were generally positively correlated with these indicators (Figure 5).

## 4. Discussion

This work shows that the combined intervention of LNT and MN-Gup attenuates HFD-induced obesity in mice, suggesting a potential nutritional strategy for metabolic management that warrants further investigation in preclinical settings. The formulation offers a range of beneficial effects, including improvements in blood glucose homeostasis, insulin sensitivity, serum lipid profiles, hepatic steatosis, and intestinal morphology. Furthermore, these phenotypic improvements were accompanied by compositional modulation of the gut microbiota, highlighting the microbiome–host metabolic axis as a relevant target for this intervention.

Hepatic dysfunction and lipid dysregulation are central pathophysiological features of obesity [38]. Abnormal lipid metabolism results in elevated hepatic transaminases and increased levels of cholesterol and lipoproteins, the excessive buildup of which can cause endoplasmic reticulum stress and mitochondrial damage [39]. Concurrently, obese states often exhibit leptin resistance, which is characterized by hyperleptinemia and decreased central sensitivity and represents an unstable energy balance [40]. As suggested by decreased indicators of hepatic injury, enhanced serum lipid profiles, and restored leptin levels, treatment with the LNT+MN-Gup synbiotic combination successfully alleviated these metabolic disorders in the current investigation. In the present experimental setting, our synbiotic approach resulted in a comparatively greater restoration of serum lipid profiles and hepatic transaminase levels than the single-strain probiotic intervention described by Ma et al. [41], though direct cross-study comparisons should be interpreted cautiously. The observed improvements may partly result from interactions between LNT and MN-Gup, such as potential metabolic cross-feeding. As a preferred substrate, LNT dramatically increases *Bifidobacterium* activity and SCFA production, which is consistent with Ojima et al.’s theory of “prebiotic-driven niche expansion” [42]. Additionally, prior research has shown that MN-Gup can strengthen the intestinal barrier, reducing hepatic inflammation [14]. The increased levels of gut-derived signaling molecules, such as SCFAs, can effectively improve central leptin responsiveness [43]. Previous studies have shown that acetate and propionate can cross the blood–brain barrier or signal via vagal afferents to modulate hypothalamic AMP-activated protein kinase (AMPK) activity, activate POMC/CART neurons, and participate in appetite regulation [44,45]. Furthermore, our study’s treatment of hyperleptinemia raises the possibility that this synbiotic formulation could successfully combat central leptin resistance. This result is in line with earlier studies; for example, Puccio et al. [46] documented the long-term metabolic benefits of HMO interventions in infants, and Sims et al. [47] discovered that dietary interventions considerably decreased the concentrations of leptin in breast milk of overweight lactating women. Collectively, these findings demonstrate how nutritional treatments can modify hormonal signaling, including leptin pathways, to change host metabolic states. When LNT and MN-Gup are administered together, systemic protection is provided, and the host’s energy balance is restored, with a concurrent reduction in hepatic inflammation.

Hepatic lipid deposition and adipose tissue remodeling constitute core pathological hallmarks of obesity, reflecting systemic energy imbalance, excessive de novo lipid synthesis, impaired fatty acid oxidation, and oxidative stress [48]. Histopathological analyses and the measurement of metabolic markers, such as liver lipid content, antioxidant capacity, and the expression of important metabolic regulators, are usually used to assess these changes [49,50]. The present study revealed that high-dose synbiotic (LNT+MN-Gup) intervention was associated with the reversal of HFD-induced hepatic steatosis and epididymal adipocyte hypertrophy. The synbiotic intervention not only effectively cleared ectopic hepatic lipids and restored normal hepatocyte morphology but also significantly enhanced the total antioxidant capacity (SOD, GSH-Px) of liver tissues. This was accompanied by the downregulation of lipogenesis-related genes (*Srebf1*, *Fasn*) and the upregulation of the fatty acid oxidation-regulating gene (*Ppargc1a*). These findings are highly consistent with previous literature regarding the amelioration of obesity by *Bifidobacterium* and synbiotics, further highlighting the superiority of the combined intervention. For instance, Ma et al. [41] reported that solitary supplementation with *Bifidobacterium animalis* subsp. *lactis* could significantly reduce body fat accumulation and alleviate hepatic inflammation in HFD-fed mice by modulating the gut microbiota. Furthermore, research by Zhang et al. [51] explicitly indicated that a synbiotic system combining *Bifidobacterium* with conventional prebiotics exhibited superior efficacy in improving lipid metabolism disorders compared to single-component interventions. The current study suggests a promising interactive potential between LNT and MN-Gup, which aligns with previous research [52]. While probiotics and prebiotics alone can modulate the intestinal community, their combination was associated with greater substrate utilization and enrichment of beneficial metabolites, suggesting possible synergistic effects that warrant further investigation. Therefore, the “gut–liver axis,” a microbiota–host interaction mechanism, is primarily responsible for the restoration of lipid homeostasis seen in target organs in our investigation. In particular, the high quantities of SCFAs and other metabolites generated by synbiotic fermentation probably operate as crucial signaling molecules that remotely mediate the activation of fatty acid catabolism and the repression of hepatic lipogenic pathways. SCFAs produced by microbial fermentation, particularly acetate, propionate, and butyrate, can enter the portal circulation and act as signaling molecules in the liver. SCFAs produced by microbial fermentation may contribute to the regulation of hepatic lipid metabolism, potentially via pathways such as AMPK and PPARα activation, as suggested by the literature, although these mechanisms were not directly measured in the current study. Additionally, SCFAs may engage G-protein-coupled receptors (GPR41/GPR43) to modulate hepatic lipid and glucose metabolism. They may also influence inflammatory signaling through NF-κB and cytokine pathways. Collectively, these molecular interactions provide a mechanistic basis for the observed improvements in hepatic steatosis and serum lipid profiles following LNT+MN-Gup intervention, linking microbial metabolite production to host lipid homeostasis. However, it must be transparently noted that while these protein-level signaling cascades (such as AMPK and GPR41/GPR43 activation) provide a highly plausible literature-supported framework, they were not directly measured in our current study and warrant further experimental validation.

As important drivers of host metabolic homeostasis, structural alterations in intestinal morphology and barrier integrity are widely acknowledged as the main causes of obesity-associated systemic low-grade inflammation [53,54]. The evaluation of these changes usually entails measuring molecular markers such as tight junction proteins, mucins, and pore-forming proteins like *Cldn2*, as well as assessing epithelial structural integrity by histology [55,56,57]. The current study found that consumption of a high-fat diet was linked to significant villus damage and altered expression of important genes connected to the barrier. The high-dose synbiotic treatment was associated with the greatest restoration of colonic structural integrity compared to LNT or MN-Gup alone, which produced partial improvements. Tight junction protein and mucin levels increased, *Cldn2* expression decreased, and histological morphology showed improvement with reduced inflammatory infiltration, suggesting a potential protective effect, although direct functional measurements of barrier integrity were not performed. As suggested by Liao et al. [58], the observed trend toward greater effectiveness of the synbiotic over its constituent parts may reflect complementary benefits of this combined formulation, although direct functional measurements of barrier integrity (e.g., permeability assays) were not performed in this study. In particular, *Bifidobacterium* strains aid in barrier repair by producing SCFAs and influencing immune responses [59], while HMOs like LNT are known to support the epithelium nutritionally and increase mucin production [60]. Therefore, this combination of improved epithelial adherence, optimized microbial fermentation, and immune control is likely responsible for the notable recovery of structural markers related to epithelial integrity.

A major aspect linked to obesity is the disruption of the gut microbiota, which is crucial in controlling host metabolism [61,62,63]. While changes in phylum-level community structure are frequently a sign of weakened resilience, decreased microbial richness and diversity generally imply ecological instability [64]. This study found that high-fat diet feeding was associated with different structural changes in the gut microbiota and a significant reduction in α-diversity. The high-dose synbiotic formulation was linked to the greatest restoration of microbial equilibrium, even when therapies using LNT or MN-Gup alone only produced partial responses. A normalization of community structure and a notable alteration of particular bacterial taxa associated with metabolic health served as proof of this. The observed alterations in particular bacterial species are consistent with the body of research on dysbiosis associated with obesity. For example, the enrichment of *Akkermansia*, *Leuconostoc*, and *Alistipes*, which have anti-inflammatory and barrier-strengthening qualities, is linked to better metabolic outcomes [65,66,67,68]. Concurrently, the restoration of *Muribaculaceae* and *Ileibacterium*, genera known for their roles in carbohydrate metabolism and SCFA production, further supported these beneficial effects [69,70,71,72,73]. On the other hand, the decrease in Desulfovibrionaceae_unclassified, *Erysipelatoclostridium*, *Colidextribacter*, *Helicobacter*, *Peptococcaceae_unclassified*, and *Firmicutes_unclassified*, which are often linked to lipogenesis [74,75,76], indicates a mitigation of obesogenic microbial characteristics. Interestingly, compared to the HFD group and the single-component therapies, the high-dose synbiotic intervention was linked to noticeably greater amounts of fecal SCFAs, such as acetate, propionate, and butyrate. This improvement is probably the result of a specific substrate-driven interaction: LNT functions as an exclusive nutritional niche [77] that robustly drives the expansion of the *Bifidobacterium* genus. Although 16S sequencing cannot resolve strain-level dynamics, this pronounced genus-level enrichment is consistent with, but does not directly demonstrate, the potential in vivo persistence and metabolic activation of the administered MN-Gup strain, facilitating the robust production of SCFAs [16], especially acetate and propionate, though direct strain-level colonization would require future metagenomic validation. Consistent with these metabolite changes, PICRUSt2-based functional prediction indicated that the intervention primarily affected metabolism-related pathways in the gut microbiota. Among these, lipid metabolism was one of the most prominent functional categories altered across groups, while energy metabolism, nucleotide metabolism, metabolism of cofactors and vitamins, and glycan biosynthesis and metabolism also showed corresponding changes. Together with the increased SCFA levels, these results suggest that the synbiotic intervention was associated with a shift in gut microbial function toward a metabolic profile more favorable for host metabolic homeostasis.

Spearman’s correlation analysis revealed associations between the high-dose synbiotic intervention and changes in multiple metabolic tissues. The intervention shifted the gut microbiota from an obesogenic to a healthier profile, marked by the enrichment of beneficial taxa such as *Akkermansia*, *Leuconostoc*, *Alistipes*, and *Muribaculaceae*, and the suppression of potentially obesogenic genera including *Desulfovibrionaceae_unclassified*, *Erysipelatoclostridium*, and *Colidextribacter*. Beneficial bacteria were positively correlated with hepatic fatty acid oxidation (e.g., *Ppargc1a*) and antioxidant capacity (SOD, GSH-Px), and negatively correlated with lipogenic genes (*Srebf1*, *Fasn*) and hepatic lipid accumulation. In contrast, obesogenic taxa correlated positively with hepatic steatosis and adipocyte hypertrophy. Furthermore, our analysis successfully linked these microbial shifts to enhanced SCFA production. Specifically, beneficial taxa such as *Akkermansia* showed a positive correlation with butyrate levels, and *Muribaculaceae* correlated positively with acetate and propionate. Conversely, opportunistic genera like *Colidextribacter* exhibited a negative correlation with butyrate. These relationships are consistent with previous studies: *Akkermansia* enhances gut barrier integrity, fortifies mucosal defense against inflammatory triggers, and promotes hepatic fatty acid catabolism [78]; *Alistipes* and *Muribaculaceae* produce SCFAs that activate hepatic AMPK and PPARα signaling, improving lipid metabolism [79]; whereas *Desulfovibrionaceae* and *Erysipelatoclostridium* produce proinflammatory metabolites that exacerbate hepatic lipogenesis and adipose expansion [80]. Collectively, these correlations suggest potential links between gut microbiota remodeling and metabolic changes in the liver and adipose tissue, which may be consistent with the proposed gut–liver and gut–adipose axes. The enhanced microbial profile was associated with improved markers of intestinal barrier integrity, including *Cldn2* and *Muc2* expression, suggesting a potential contribution to restricting the leakage of luminal antigens. These associations may underlie the observed improvements in obesity-related metabolic parameters following high-dose synbiotic treatment, although causal relationships remain to be established.

In summary, the present study demonstrates that a targeted synbiotic formulation comprising LNT and MN-Gup exerts beneficial protective effects on intestinal structural integrity, hepatic glucolipid metabolism, gut microecology, and systemic energy homeostasis. These comprehensive improvements contribute to mitigating HFD-induced obesity and associated metabolic dysfunctions. The complex multi-organ crosstalk was further substantiated by Spearman’s correlation analysis, which revealed robust associations between specific microbial taxa and key host metabolic indices. Overall, these findings advance our understanding of microbiota-targeted metabolic interventions and provide an initial experimental basis for further preclinical exploration of anti-obesity strategies. Despite these findings, several critical limitations must be explicitly acknowledged. First, given the intrinsic physiological and microbiological disparities between species, extrapolating findings from murine models to human clinical outcomes requires caution. Second, the experiments were conducted exclusively in male mice. While males provide a robust and consistent model for high-fat diet-induced obesity, this single-sex design precludes the assessment of sex-specific metabolic responses. Third, although we observed local improvements in histological morphology and barrier-related gene expression, we lacked direct functional measurements of intestinal permeability and systemic indicators of metabolic endotoxemia, such as circulating LPS. Fourth, our microbiota analysis relied on 16S rRNA sequencing and targeted SCFA quantification; the absence of shotgun metagenomics and untargeted metabolomics restricts deeper functional validation. Consequently, we were unable to directly track the colonization of the MN-Gup strain at the strain level within the host gut. Furthermore, it remains to be explored whether this highly specific substrate-driven efficacy can be replicated with other prebiotic/probiotic combinations. Future investigations incorporating multi-omics approaches, direct functional assays, and dual-sex cohorts are essential to fully decipher these underlying mechanisms and rigorously evaluate the translational potential of this formulation in human clinical trials.

## 5. Conclusions

In summary, this study demonstrates that the synbiotic composed of LNT and MN-Gup exhibits superior anti-obesity efficacy compared to single-component interventions. Precisely distinguishing its metabolic effects revealed that this targeted formulation distinctly promotes a more robust in vivo enrichment of *Bifidobacterium* and enhances SCFA production than either component alone. This synbiotic approach promotes comprehensive and coordinated benefits across multi-tissue axes, including microbial remodeling, intestinal barrier preservation, hepatic metabolic regulation, and systemic energy homeostasis. Ultimately, our findings clearly indicate that synbiotic modulation of the gut microbiota serves as an effective therapeutic strategy for mitigating obesity and associated metabolic diseases.

## Figures and Tables

**Table 1:** Primer sequences used in qPCR assays.

Gene	Organism	Sequence 5′-3′	Annealing T °C
*Gapdh*	Mouse	F:TGACCTCAACTACATGGTCT	55
		R:CTTCCCATTCTCGGCCTTG	
*Srebf1*	Mouse	F:TATGGAGGGCATGAAACCCGAAGT	60
		R:TTGACCTGGCTATCCTCAAAGGCT	
*Fasn*	Mouse	F:GCAGCAAGTGTCCACCAACAA	60
		R:CTCATCGGAGCGCAGGATAGA	
*Acaca*	Mouse	F:TAACAGAATCGACACTGGCTGGCT	60
		R:ATGCTGTTCCTCAGGCTCACATCT	
*Pparg*	Mouse	F:TTTTCAAGGGTGCCAGTTTC	55
		R:AATCCTTGGCCCTCTGAGAT	
*Ppargc1a*	Mouse	F:GCAGCGGTCTTAGCACTCAGAAC	60
		R:GGAGGGTCATCGTTTGTGGTCAG	
*Cyp7a1*	Mouse	F:TCAAAGAGCGCTGTCTGGGTCA	60
		R:TTTCCCGGGCTTTATGTGCGGT	
*Cd36*	Mouse	F:ATGGGCTGTGATCGGAACTG	60
		R:GTCTTCCCAATAAGCATGTCTCC	
*Tjp1*	Mouse	F:GCCGCTAAGAGCACAGCAA	60
		R:TCCCCACTCTGAAAATGAGGA	
*Cdh1*	Mouse	F:CTCCAGTCATAGGGAGCTGTC	60
		R:TCTTCTGAGACCTGGGTACAC	
*Muc2*	Mouse	F:GTCCTGACCAAGAGCGAACA	59
		R:ACAGCACGACAGTCTTCAGG	
*Cldn2*	Mouse	F:CAACTGGTGGGCTACATCCTA	60
		R:CCCTTGGAAAAGCCAACCG	
*Ocln*	Mouse	F:TGGCTATGGAGGCGGCTATGG	60
		R:AAGGAAGCGATGAAGCAGAAGGC	

## Data Availability

The original contributions presented in this study are included in the article/Supplementary Materials. Further inquiries can be directed to the corresponding authors.

## Supplementary Materials

The following supporting information can be downloaded at: https://www.mdpi.com/article/10.3390/nu18111681/s1, Table S1: The composition of the 60% high-fat diet; Figure S1: Observed species rarefaction curves; Figure S2: Concentrations of other SCFAs with no significant changes: (A) isobutyrate, (B) valerate, and (C) isovalerate; Figure S3: Spearman correlation analysis between bacterial genus and basic indicators.

## Abbreviations

The following abbreviations are used in this manuscript:

HFD	High-fat diet
FBG	Fasting blood glucose
IPGTT	Intraperitoneal glucose tolerance test
TG	Total triglyceride
TC	Total cholesterol
ALT	Alanine aminotransferase
AST	Aspartate aminotransferase
HDL-C	High-density lipoprotein cholesterol
LDL-C	Low-density lipoprotein cholesterol
LEP	Leptin
T-SOD	Total superoxide dismutase
GSH-PX	Glutathione peroxidase
H&E	Hematoxylin and eosin
ORO	Oil Red O staining
16S rRNA	16S ribosomal RNA
NC	Normal control
PBS	Phosphate-buffered saline
LNT	Lacto-*N*-tetraose
MN-Gup	*Bifidobacterium animalis* subsp. *lactis* MN-Gup
LPS	Lipopolysaccharide
SCFAs	Short-chain fatty acids
GLP-1	Glucagon-like peptide-1
HMOs	Human milk oligosaccharides
MRS	De Man, Rogosa, and Sharpe
ELISA	Enzyme-linked immunosorbent assay
PFA	Paraformaldehyde
LEfSe	Linear discriminant analysis effect size
NMDS	Non-metric Multidimensional Scaling
QIIME2	Quantitative Insights Into Microbial Ecology 2
DADA2	Divisive Amplicon Denoising Algorithm 2
NCBI	National Center for Biotechnology Information
PICRUSt2	Phylogenetic Investigation of Communities by Reconstruction of Unobserved States 2
