# Supplementary material for: A Synbiotic of Lacto-N-tetraose and Bifidobacterium animalis subsp. lactis MN-Gup Attenuates High-Fat Diet-Induced Obesity by Modulating Metabolism and Gut Microbiota in Mice
Source: Nutrients. 2026 May 24;18(11):1681. doi: 10.3390/nu18111681 (PMC13258041; doi:10.3390/nu18111681)

Supplementary materials

Table S1. The composition of the 60% high-fat diet.

60% High-fat diet	
Ingredient	Content (g/kg)
Casein	200.00
L-Cystine	3.00
Corn Starch	0
Maltondextrin 10	125.00
Sucrose	72.80
Cellulose	50.00
Soybean Oil	25.00
Lard	245.00
Mineral mix S10026B	50.00
Vitamin Mix V10001C	1.00
Choline Bitartrate	2.00
FD&C Red Dye#40	0
FD&C Yellow Dye#5	0
FD&C Blue Dye#1	0.05
Total	773.85

Figure S1. Observed species rarefaction curves.

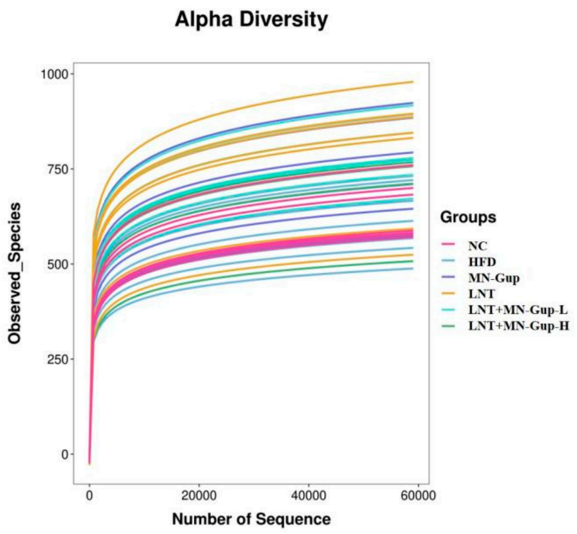

Figure S2. Concentrations of other SCFAs with no significant changes: (A) isobutyrate, (B) valerate, and (C) isovalerate.

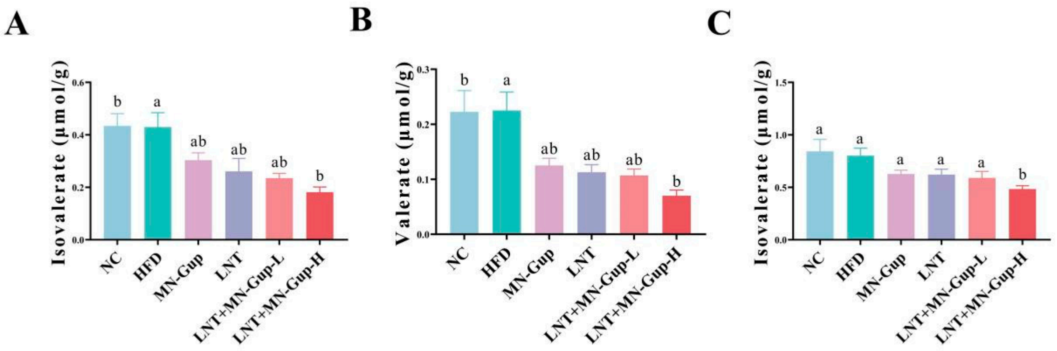

Figure S3. Spearman correlation analysis between bacterial genus and basic indicators. Correlations were considered statistically significant when $*p < 0.05$, $**p < 0.01$, and $***p < 0.001$.

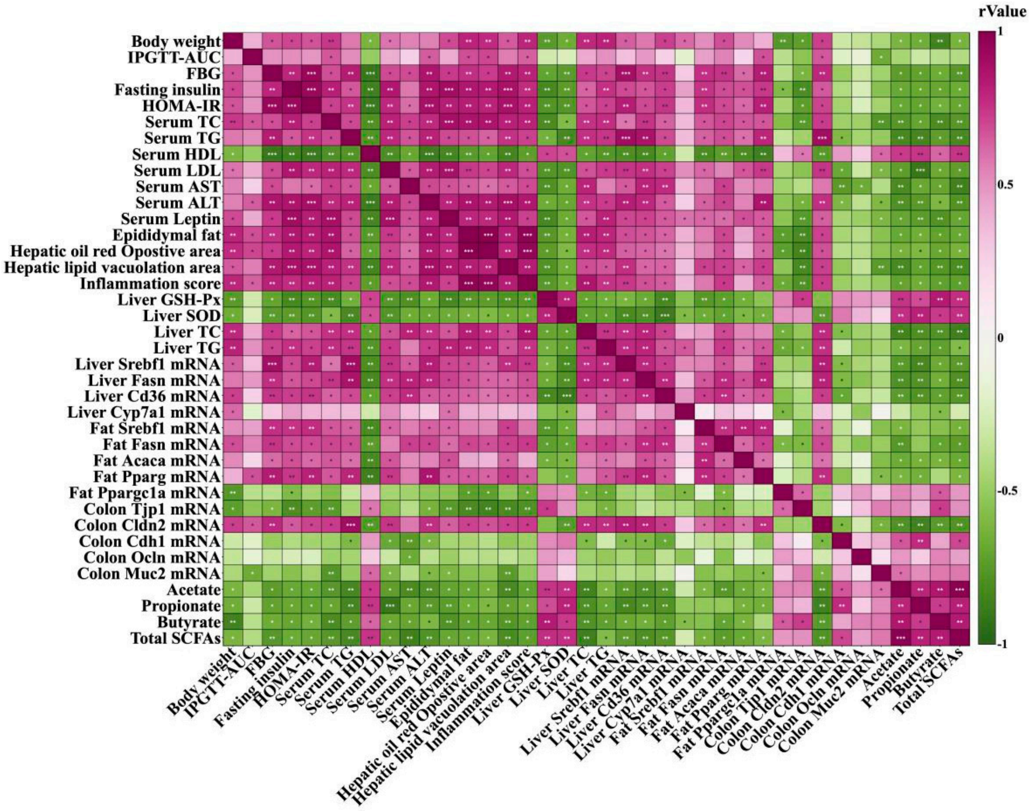

Supplement: Supplementary file 1 [file nutrients-18-01681-s001.zip › nutrients-4274765-supplementary.pdf]
